# Too Good to Keep—Differential Diagnosis

**Published:** 1896-11

**Authors:** 


### Correspondence.

---

### Too Good to Keep—Differential Diagnosis.

A valued correspondent writes as follows:

Dear Dr. Daniel:—I am bonnd to tell you the latest, which occurred here a few days ago. I give it to you as it came to me.

The incident I am about to relate to you, while very amusing and laugh provoking, might have been a very serious matter for at least one of the parties concerned, had not one of our "old fogy doctors" (as our youthful M. D.'s are pleased to denominate us old fellows) stepped in and broke up the game.

You know Dr. B. well, and you know him to be an easy-going fellow, perfectly original in his ways. Ready and willing at all times to extend the hand of fellowship to all, both the "new" and the "old." Ready at all times, night or day, to help a brother *Medico* out of trouble, which has made him exceedingly popnlar, especially with us old fellows. The *dramatis personæ* in this comedy of errors were, two ladies, three young M. D's. and the said "old fogy doctor." A short time since, a lady from up about *somewhere* came to our city to consult some one of our eminent resident surgeons in regard to a very prominent condition of her abdomen. In the neighborhood where she obtained board a youthful fledgling of the immortal Galen had pitched his tent, and at the suggestion of the landlady, he was at once summoned. After a careful and thorough examination, the doctor said "ovarian tumor." To confirm his diagnosis two other young physicians were called in, who, after viewing the case, confirmed the opinion of their colleague. An operation was decided upon. A day was set and all preparations made for the event. During the interim the lady wrote to her sister stating the opinion of the doctors, and their determination to make the operation on a certain day. The sister at once left her home and hastened to our city, determined to be with her relative in her sore distress. When the appointed day came for operating, the physicians came with it, loaded with all the paraphernalia requisite for the matter in hand. The visiting lady regarded the youthful trio, and addressing them, asked what experience they had had in making the operation which they were about to undertake. They said that their experience was limited to the description of the operation, as presented in their works on surgery, but that they felt entirely competent to make the operation. "Well, gentlemen," said the visiting lady, "is there no physician in ——— who has made this operation?" Not to their knowledge, they said. "Then, in that case," said the lady, we will defer this matter to some future day; this is the only sister I have, and I don't care to run any risks." So ended the second act in our comedy.

One or two days subsequent to the foregoing incidents, a visiting friend of the family called in, and the circumstances of the case being related to her, she said she knew of a physician who had made the operation a number of times, and successfully, she thought, in every instance, and mentioned the name of Dr. B. as the surgeon who had made the operation. Dr. B. was at once sent for; the case was presented to him with all the details in regard to the proposed operation. After a thorough understanding in regard to the case, Dr. B. proceeded to make his examination. As the examination progressed there grew on the face of the doctor an amused expression, which gradually broadened into a smile, as the reverberation sounds elicited by percussion filled the room, clear and resonant in tone, the evidence was unmistakable. Turning to the ladies the doctor remarked, "Let me make the operation in this case," they readily assented. The doctor said that it would be necessary to give a little medicine preparatory to operating, and with this he handed the lady a prescription with directions in regard to taking it. In a short time the patient began to pass great quantities of gas, the tumor grew less and less in size, until after a period of twenty-four hours the woman was as flat as a board. Comment is unnecessary. So much for the "old fogy doctors."

Yours truly,

